# Positive feedback between peanut and arbuscular mycorrhizal fungi with the application of hairy vetch in Ultisol

**DOI:** 10.3389/fmicb.2022.1002459

**Published:** 2022-09-26

**Authors:** Xingjia Xiang, Jinyi Zhang, Guilong Li, Ke Leng, Luyuan Sun, Wenjing Qin, Chunrui Peng, Changxu Xu, Jia Liu, Yuji Jiang

**Affiliations:** ^1^School of Resources and Environmental Engineering, Anhui University, Hefei, China; ^2^Anhui Province Key Laboratory of Wetland Ecosystem Protection and Restoration, Hefei, China; ^3^Soil and Fertilizer and Resources and Environment Institute, Jiangxi Academy of Agricultural Sciences, Nanchang, China; ^4^Key Laboratory of Acidified Soil Amelioration and Utilization, Ministry of Agriculture and Rural Affairs, Nanchang, China; ^5^State Key Laboratory of Soil and Sustainable Agriculture, Institute of Soil Science, Chinese Academy of Sciences, Nanjing, China

**Keywords:** AMF community, hairy vetch, soil fertility, peanut yield, sequencing

## Abstract

Multiple agricultural practices are being applied to increase crop yield in order to overcome the food shortage. Green manure has emerged as an appropriate practice to improve soil fertility and crop yield. However, the potential functions of arbuscular mycorrhizal fungi (AMF) in the below-ground ecosystems following the application of green manure in Ultisols remain largely unexplored. In this study, qPCR and high-throughput sequencing were used to investigate the response of AMF abundance and communities in different treatment groups, i.e., control (without fertilization), mineral fertilization (NPK), mineral fertilization with returning peanut straw (NPKS), and with green manure (hairy vetch; NPKG). The NPKG treatment significantly increased soil fertility compared to other treatment groups. Compared with control, the NPK, NPKS, and NPKG treatments increased peanut yield by 12.3, 13.1, and 25.4%, respectively. NPKS and NPKG treatments significantly altered the AMF community composition decreased the AMF diversity and increased AMF abundance compared to the control. The AMF network of the NPKG treatment group showed the highest complexity and stability compared to other treatment groups. The structural equation modeling revealed that the application of hairy vetch improved soil nutrients and peanut yield by increasing the soil AMF abundance and network stability. Overall, the results suggested that the application of hairy vetch might trigger positive feedback between the peanut and AMF community, contributing to fertility and yield improvement in the dryland of Ultisol.

## Introduction

Ultisol, one of the most important soil types, occupies approximately 21% land area of China. Over time, high-strength and long-term farming in Ultisol have adversely impacted the ecosystem in the form of soil acidification, fertility depletion, and a decline in crop yield (Xiang et al., 2020). However, the use of organic materials (e.g., animal excreta) in agriculture practices effectively alleviates these adverse effects. The application of animal excreta to agricultural soil increased soil fertility and suppressed plant pathogens (Shahbaz et al., 2017). However, deleterious effects of animal excreta application, such as the enrichment of heavy metals and residual antibiotics have gained increasing attention (Sager, 2007; Yan et al., 2022). Thus, suitable and sustainable agricultural practice in Ultisol demands urgent investigation.

Crop straw and green manure are important renewable organic resources (Zhou et al., 2019). Maize and rice straw returning improved soil nitrogen content (Akhtar et al., 2018) and contributed to the accumulation of soil organic carbon (Lee et al., 2020; Dutta et al., 2022). However, the direct return of peanut straw to the field might result in soil-borne diseases and obstacles in continuous cropping (Li et al., 2014). Thus, the effects of returning composted peanut straw to the agroecosystem should be investigated further. Peanuts are planted in mid-late April and harvested at the end of August in South China. Monoculture is commonly practiced in Ultisols due to severe seasonal drought. The 8-month peanut planting interval provides enough time and space for planting green manure (Xiang et al., 2021). Planting green manure triggered long-term effects such as improving soil quality, and immediate effects such as the increasing supply of available nutrients for subsequent crops (Mohanty et al., 2020; Ansari et al., 2022).

Soil microbes are involved in several functions in agroecosystems, such as nutrient availability, pathogen control, and resilience to abiotic stresses (de Vries et al., 2020; Chen et al., 2021). The application of crop straw and green manure to the field triggered changes in soil microbial diversity and biomass (Xiang et al., 2021). Besides, the application of green manure improved soil fertility and crop production by activating the functions of below-ground soil microbes (Khan et al., 2020). Recently, microbial network analysis was particularly useful to statistically identify the key microbial taxa that regulate the microbial functions in the below-ground ecosystem (Banerjee et al., 2018; Yuan et al., 2021). However, few studies have explored key taxa involved in the regulation of agroecosystem using network analysis following returning crop straw and planting green manure.

Arbuscular mycorrhizal fungi (AMF) are present in the roots of plants and live in mutualistic symbiosis with most plants. AMF also improved the absorption of nutrients for plants in exchange for carbohydrates (Smith and Read, 2008). In addition, AMF improved the soil structure and increased ecosystem functions by decreasing nutrient loss from leaching (van der Heijden, 2010; Martin et al., 2012). Mineral fertilization directly increased soil fertility to decrease the dependence of plants on their AMF partners. This phenomenon might shift the relationship between the plants and AMF from mutualism to parasitism (Kiers et al., 2011). However, the previous study has shown that the addition of pig manure increased the AMF biomass (Jiang et al., 2020) and richness (Liu et al., 2020). However, the response of the AMF communities to the application of green manure and crop straw remains largely unknown in the Ultisol.

In this study, we evaluate the effects of green manure and peanut straw on soil fertility, crop yield, and AMF communities in the dryland of Ultisol using qPCR and sequencing (Illumina MiSeq). Hairy vetch (*Vicia villosa Roth* L.) was selected as the experimental green manure. Peanut straw was composted for 8 months and then returned to the field. This study aimed to investigate the effects of (a) composted straw and green manure on soil fertility and peanut yield, (b) composted straw and green manure on the AMF communities and key taxa within the AMF network, and (c) feedback among AMF, soil fertility, and peanut yield following different agricultural practices in Ultisol.

## Materials and methods

### Experimental design

The experimental site is located at the Comprehensive Experimental Station of red soil (28°10′59″N, 106°35′11″E, 50 m), Dongxiang County, Jiangxi Province, China. The soil type was classified as Ultisol. This region experiences a typical subtropical warm and humid monsoon climate. The annual average temperature and precipitation are 18°C and 2,180 mm, respectively. This experiment was established in 2014. The soil properties before the experiments were as follows: 5.34 pH, 7.69 g kg^–1^ organic C, 1.02 g kg^–1^ total N, 85.9 mg kg^–1^ alkaline N, 0.68 g kg^–1^ total P, and 12.7 mg kg^–1^ available *P*.

The experiments included four treatment groups with six replicates, i.e., (a) without fertilizer (control), (b) mineral fertilization (NPK), (c) mineral fertilization with returning composted peanut straw (NPKS), and (d) mineral fertilization with planting green manure (NPKG). Peanut straw was removed in the control, NPK, and NPKG treatment groups. Composted peanut straw was plowed into the soil 2 weeks before the next peanut planting. Dry straw is about 1,300 kg ha^–1^yr^–1^ in NPKS treatment. Green manure was applied at a seeding rate of 20 kg ha^–1^ in mid-October every year with a yield of around 2 × 10^4^ kg ha^–1^yr^–1^ in NPKG treatment. The green manure was cut into 5 cm small pieces and plowed into the soil 2 weeks before the next peanut planting. The field site was used to cultivate peanuts (cv. Yueyou 256). Peanuts were sown in a row at a spacing of 40 cm and a whole spacing of 20 cm. Two seeds were planted in each cave, and the planting density was about 1.25 × 10^5^ points hm^–2^. The peanut seedlings were planted in mid-April, and they ripened in mid to late August.

The application rates of mineral fertilizers to peanuts were: 135 kg ha^–1^ urea (containing 46.4% N), 81 kg ha^–1^ calcium superphosphate (containing 12.0% P_2_O_5_), and 135 kg ha^–1^ potassium chloride (containing 60.0% K_2_O). The 50% of N and K fertilizers were broadcasted as basal application, while the other 50% was applied at the peanut needle stage. All *P* fertilizers were applied as basal fertilizers. Basal fertilizer was thoroughly incorporated into the soil by plowing one day before the peanut plantation.

### Soil sampling

Rhizospheric soils were sampled at the pod stage on 15 July 2017. One composite rhizospheric soil sample was taken from each plot consisting of five randomly selected peanut plants. After gently pulling out the peanuts, we tap the entire root system to dislodge all the loose soil and the remaining soil was collected as a rhizospheric soil sample (Smalla et al., 2001).

### Soil chemical properties analyses

The soil water suspension (1:2.5 wt/volume) was shaken for 30 min, and then the pH of the soil water solution was determined. The potassium dichromate oxidation method was used to determine soil organic carbon (SOC). Total nitrogen (TN) was determined using an elemental analyzer (multi-EA 5,000, Jean, Germany). The molybdenum blue colorimetry method was used for measuring total phosphorus (TP) (Bowman, 1988). Alkaline nitrogen (AN) (Ai et al., 2017) was determined using the alkaline hydrolysis diffusion method. Available phosphorus (AP) (Stahlberg, 1980) was extracted using sodium bicarbonate and AP content was determined using the molybdenum blue colorimetric method.

### DNA extraction

The soil DNA was extracted from 0.5 g of soil using the Fast DNA^®^ SPIN Kit (MP Biomedicals, Santa Ana, CA, USA) according to the manufacturer’s instructions.

### Quantitative real-time PCR for arbuscular mycorrhizal fungi abundance

Primer pair AMV4.5NF/AMDGR were used to determine AMF abundance by CFX96 Optical Real-Time Detection System (Bio-Rad, Laboratories Inc., Hercules, CA, USA). Each PCR reaction was carried out in a 25 μl qPCR reaction mixture containing 12.5 μl SYBR^®^Premix Ex Taq (TliRNaseH Plus, 2×, Takara Bio, Japan), 0.5 μl PCR forward and reverse primers (both 10 μM), 2 μl DNA template, and 9.5 μl double distilled water (ddH_2_O). Quantitative real-time PCR reactions were set to 95°C for 5 min, followed by 35 cycles of 95°C for 30 s, 58°C for 30 s, and 72°C for 1 min. The melting curve analysis showed excellent specificity of the qPCR with an efficiency of 98.5% (*R*^2^ = 0.9997).

### PCR and amplicon library preparation

The PCR was performed in a 50-μL reaction mixture and the mixture contained a concentration of 1.25 mM deoxynucleoside triphosphate, 15 μM of forward and reverse primers (i.e., AMV4.5NF/AMDGR) in each, 2 U of Taq DNA polymerase (TaKaRa, Japan). Each PCR reaction mixture contained 1 μl of DNA as a template. The cycling parameters were as follows: 35 cycles of 95°C for 45 s, 58°C for 45 s, and 72°C for 1 min, with a final extension at 72°C for 10 min.

### Bioinformatics analysis

Raw data were processed using qiime2-2020.2 (Bolyen et al., 2019). The deblur algorithm was used to filter poor-quality sequences (Amir et al., 2017). Then, the chimeras of amplicon sequence variants (ASVs) were filtered using vsearch (Rognes et al., 2016). All singleton ASVs were removed. Annotations for ASV were conducted by classify-sklearn using the MaarjAM database (Öpik et al., 2010). A subset of 21,000 sequences was randomly selected per sample for further analysis.

### Statistical analysis

The differences in the AMF index (i.e., AMF abundance, AMF richness, and the dominant taxa) and soil properties among different treatments were tested using one-way ANOVA. Canonical analysis of principal coordinates (CAP), ANOSIM, and RDA analyses were conducted using the R v.4.0.4 software. The primary factors affecting AMF abundance, diversity, and relative abundance of dominant taxa were determined using the Random Forest model (Trivedi et al., 2016). The differences in AMF species were detected using STAMP 2.1.3 software among treatment groups. The co-occurrence network was performed using high-frequency ASVs (>3 samples) with robust (Spearman’s *r* > 0.6 or <−0.6), and significant (*P* < 0.05) Benjamini–Hochberg multiple testing correction. Topological features (i.e., average degree, natural connectivity, and the average clustering coefficient) were determined using the igraph package in R v.4.0.4 software. The structural equation modeling (SEM) was performed using the lavaan package in R v.4.0.4 software to assess the direct and indirect contributions of abiotic (soil nutrient) and biotic (AMF index) variables to peanut yield.

## Results

### Soil properties and peanut productivity

Different agricultural practices triggered significant changes in the soil properties (Table 1). The soil pH in NPKS and NPKG treatment groups was decreased to 4.97 and 4.71, respectively, compared to control (pH 5.21). Besides, the NPKG treatment significantly increased soil fertility levels. Compared to control, peanut yield was significantly increased by 12.3% in NPK, 13.1% in NPKS, and 25.4% in NPKG (Table 1).

**TABLE 1:** The summary of the main soil properties and peanut yield.

Variables	Control	NPK	NPKS	NPKG
Soil pH	5.21 ± 0.10a	5.29 ± 0.17a	4.97 ± 0.14b	4.71 ± 0.20c
Soil organic carbon (g kg^–1^)	7.82 ± 0.30b	7.64 ± 0.37b	8.00 ± 0.47b	10.6 ± 0.37a
Total nitrogen (g kg^–1^)	0.99 ± 0.04b	0.95 ± 0.05b	0.96 ± 0.05b	1.20 ± 0.05a
Total phosphorus (g kg^–1^)	0.67 ± 0.02b	0.74 ± 0.06ab	0.67 ± 0.10b	0.75 ± 0.03a
Alkaline nitrogen (mg kg^–1^)	86.4 ± 4.50b	90.0 ± 2.01b	91.3 ± 2.77b	128 ± 16.8a
Available phosphorus (mg kg^–1^)	13.1 ± 1.68bc	16.0 ± 3.06b	11.9 ± 2.69c	23.4 ± 2.18a
Peanut yield (kg ha^–1^)	2864 ± 192.9c	3217 ± 174.3b	3240 ± 224.7b	3591 ± 278.3a

The values in brackets represent the standard deviation of the mean. Letters following numbers represent significant differences from one-way ANOVA with Duncan’s HSD comparisons (*P* < 0.05).

### Soil arbuscular mycorrhizal fungi abundance and alpha-diversity

**FIGURE 1:**
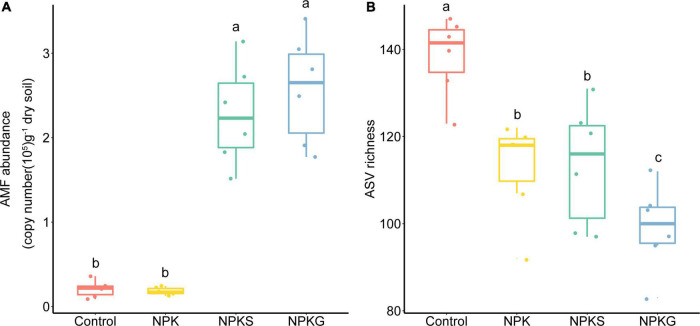
The variation of arbuscular mycorrhizal fungi (AMF) abundance **(A)** and ASV richness **(B)** in the soil among different treatments. The bottom and top of the box denote the first and third quartiles; the band inside the box denotes the median. Letters represent significant differences from the one-way ANOVA with Tukey’s HSD comparisons (*P* < 0.05).

The NPKS and NPKG treatments increased AMF abundance by 10.9 times and 12.2 times, respectively, while NPK treatment had little effect on the AMF abundance compared to the control (Figure 1A). The AMF abundance was significantly correlated with the soil pH, SOC, and AN (Supplementary Figure 1). Compared with the control, the NPK, NPKS, and NPKG treatments significantly decreased the AMF richness, and the lowest AMF richness was observed in the NPKG treatment group (Figure 1B). The ASV richness was significantly correlated with the soil pH and soil nutrient contents (Supplementary Figure 1).

### Soil arbuscular mycorrhizal fungi community composition

**FIGURE 2:**
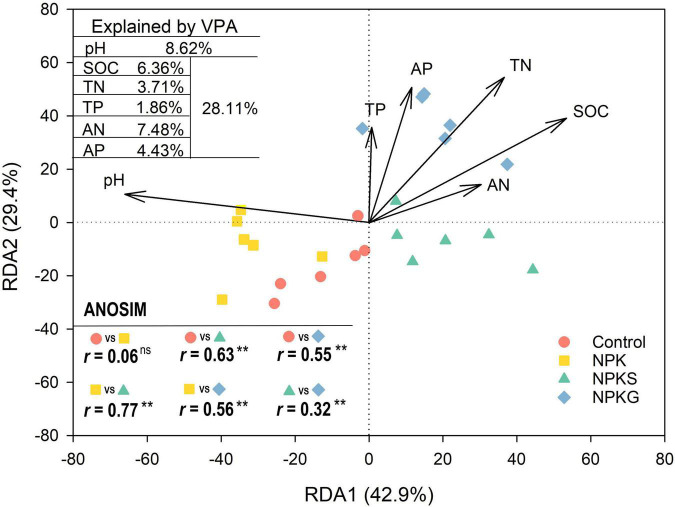
Redundancy analysis (RDA) plot showing AMF community composition across different treatments. Differences in the soil AMF community composition among different treatments were determined using Analyses of Similarities (ANOSIM). The explanation rate for each factor is placed in the upper left corner. SOC, soil organic carbon; TN, total nitrogen; AN, alkaline nitrogen; AP, available phosphorus; and TP, total phosphorus. **P < 0.01.

The organic application significantly altered the soil AMF community composition. However, mineral fertilization had little impact on the AMF community composition (Figure 2 and Supplementary Figure 2). The shifts in the AMF community composition were driven by practice-mediated changes in the soil pH and nutrients (Figure 2). At the order level, compared to control, the NPK and NPKG treatments decreased and increased the relative abundance of Glomerales, respectively (Supplementary Figure 3). The NPKG treatment decreased the relative abundances of Diversisporales and Paraglomerales compared to the control (Supplementary Figure 3). At the family level, compared to the control, the NPKS and NPKG treatments significantly increased the relative abundance of Glomeraceae and decreased the relative abundance of Claroideoglomeraceae. The relative abundances of Acaulosporaceae and Gigasporaceae were significantly increased in the NPK treatment group and decreased in the NPKG treatment group compared to the control (Supplementary Figure 4). The STAMP analysis showed that NPKS treatment significantly decreased the relative abundance of *Claroideoglomus*, and NPKG treatment significantly increased the relative abundance of *Glomus* and decreased the relative abundance of *Claroideoglomus* and *Acaulospora* (Supplementary Figure 5).

### Characteristics of co-occurrence networks

**FIGURE 3:**
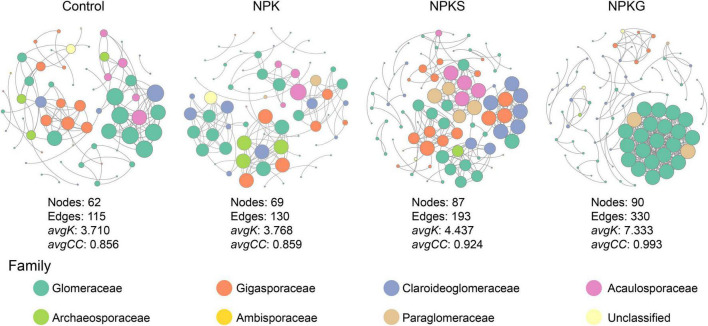
Co-occurrence AMF network for each treatment. Each node in the figure represents an amplicon sequence variant (ASV), and the size of the point represents the degree of the node.

The soil AMF network was investigated for each agricultural practice separately in this study (Figure 3). The lower number of nodes (e.g., taxa) and edges (interactions among taxa) were found in the networks of control, NPK, and NPKS treatment groups. The network of NPKG consisted of a higher number of nodes and edges. The average degree and the average clustering coefficient in the NPKG network were also considerably higher than in the other three networks. The NPKG network exhibited higher robustness and stability than the other three networks (Supplementary Figure 6). Furthermore, the topological characteristics of the sub-network within Glomeraceae were also significantly higher in the NPKG treatment group than in other treatment groups (Supplementary Figure 7).

### The effect of abiotic and biotic variables on yield

**FIGURE 4:**
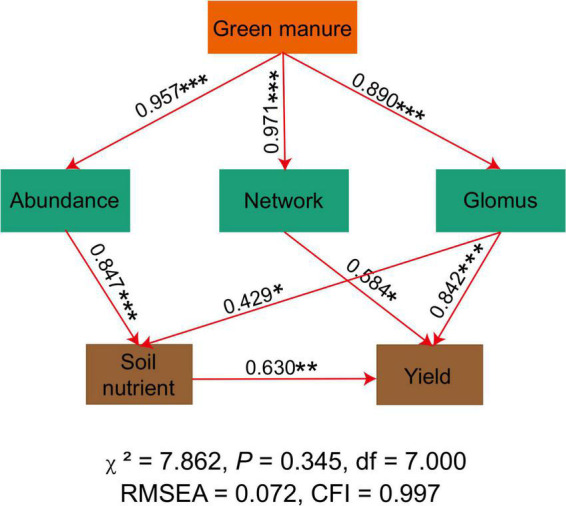
The structural equation model shows the relationships among AMF index, soil nutrient, and crop yield. The solid line represents a significant relationship, and the dotted line represents an insignificant relationship. *, **, and *** in the figure represents significance at the probability of 0.05, 0.01, and 0.001 level, respectively. All variances attributable to the constrained factor and the significance of the factor are portrayed at the bottom of the plot.

The SEM analysis demonstrated that the application of green manure was associated with a higher AMF abundance, network complexity, and relative abundance of *Glomus* (Figure 4). Specifically, planting green manure improved the soil nutrient level indirectly by increasing the AMF abundance and the relative abundance of *Glomus*. Soil nutrients, AMF network complexity, and relative abundance of *Glomus* showed a significant and positive correlation with the peanut yield (Figure 4).

## Discussion

Previous studies have shown that the application of green manure improved fertility and yield (Mohanty et al., 2020; Ansari et al., 2022; Table 1). In this study, shifts in certain AMF taxa might be crucial to improving the yield in addition to increasing soil fertility following the application of hairy vetch (Bonanomi et al., 2020). Soil AMFs are beneficial for the absorption of soil nutrients (de Gruyter et al., 2022). The Glomeraceae showed the highest P uptake efficiency and antagonistic activities against plant pathogens (Kaur et al., 2022). However, Gigasporaceae showed a negative impact on the plant growth potential (Li et al., 2008). In this study, the application of hairy vetch increased the relative abundance of Glomeraceae and decreased the relative abundance of Gigasporaceae (Supplementary Figure 4). These results indicated that the application of hairy vetch might benefit the agricultural ecosystem by optimizing soil AMF taxa and regulating below-ground AMF functions.

The AMF could recruit soil saprotrophs to decompose organic matter following organic amendment (Xiang et al., 2015; Frey, 2019; Wang et al., 2021). Especially, the AMF genus *Glomus* might act as a conduit to transport plant-derived monosaccharides from the roots to the soil aggregates (Johnson et al., 2002; Jeewani et al., 2021), which might regulate soil saprotrophs for the transformation of organic matters. In this study, the application of hairy vetch significantly increased the relative abundance of *Glomus* (Supplementary Figure 5). This demonstrates that certain beneficial taxa (i.e., *Glomus*) might accelerate organic degradation to increase mineral fertilizers levels following the application of hairy vetch (Table 1).

Previous studies have shown the negative response of AMF diversity to nutrient enrichment (Alguacil et al., 2010; Camenzind et al., 2014; Figure 1B and Supplementary Figure 1). However, the decreased microbial diversity did not completely reflect the loss of ecological functions due to the functional redundancy of the microbes (Louca et al., 2018; Frew et al., 2022). Moreover, previous studies have shown that the primary driver of soil multifunctionality was microbial biomass/abundance rather than microbial diversity (Garland et al., 2021; Wang et al., 2021; Frew et al., 2022). In line with the previous studies, planting hairy vetch significantly increased the soil AMF abundance (Jiang et al., 2021; Figure 1A). A higher AMF abundance could result in the degradation of organic matter to release mineral nutrients for plants (Treseder and Cross, 2006; Figure 4).

The soil microbial networks reveal the microbial structure and their response to environmental fluctuations (Wagg et al., 2019; Yuan et al., 2021). This study showed that the application of hairy vetch had a significantly more intricate network than other treatments (Figure 3). Previous studies have shown that the networks with more sophisticated architecture were more stable to environmental fluctuations and might support higher ecosystem multifunctionality (Wagg et al., 2019). Thus, the application of hairy vetch might increase the complexity of the soil AMF network to strengthen below-ground multifunctionality, contributing to nutrient level and crop productivity.

In this study, the effects of returning composted straw on the soil nutrients and peanut yields were not remarkable compared to the application of hairy vetch (Table 1). The peanut straw showed a higher carbon content and lower nitrogen and phosphorus contents (Zhou et al., 2019). More nutrient loss occurred after nearly 8 months of composting. Thus, returning composted straw might not be an appropriate practice in the dryland of Ultisol. Besides, the application of the hairy vetch increased AMF abundance and optimized AMF community structure to increase soil nutrients and peanut yields (Figures 1–4 and Table 1). However, the results of this study also demonstrated that the soil pH decreased sharply following planting green manure. Thus, attention should be paid to the negative effect of soil acidification following the long-term application of green manure.

## Conclusion

This study demonstrated the differential feedback between peanut and their AMF partners in different agricultural practices. Planting hairy vetch significantly increased the AMF abundance, and network complexity, and optimized soil AMF taxa, contributing to the nutrient and yield improvement in the dryland of Ultisol. The application of hairy vetch might trigger positive feedback to strengthen the mutualism between crops and their AMF partners in the agroecosystem. In addition, returning peanut straw, without increasing nutrient levels but triggering soil acidification, might be an inappropriate practice in Ultisol. Overall, this study added to a growing body of knowledge on underlying feedback between plants and their AMF partners following different practices in Ultisol. However, there are certain limitations to this study. The AMF infection rate which could evaluate the feedback between plant and AMF was not measured. As the application of leguminous green manure increased the soil nitrogen content, we did not clarify the role of microbes within nitrogen cycling following the application of hairy vetch. These limitations should be addressed in future studies.

## Data availability statement

The datasets presented in this study can be found in online repositories. The names of the repository/repositories and accession number(s) can be found in the article/Supplementary material.

## Author contributions

JZ, JL, and XX designed the experiments. JZ, GL, KL, LS, WQ, and CP completed the field sampling. JZ, GL, and XX performed the data analysis and prepared the figures. JZ and GL wrote the manuscript. YJ, CX, JL, and XX contributed to the revision of manuscript. All authors contributed to the article and approved the submitted version.
