# Supplementary material for: Positive feedback between peanut and arbuscular mycorrhizal fungi with the application of hairy vetch in Ultisol
Source: Front Microbiol. 2022 Sep 26;13:1002459. doi: 10.3389/fmicb.2022.1002459 (PMC9549289; doi:10.3389/fmicb.2022.1002459)
Supplement: Supplementary file 1 [file Presentation_1.pdf]

Support Information

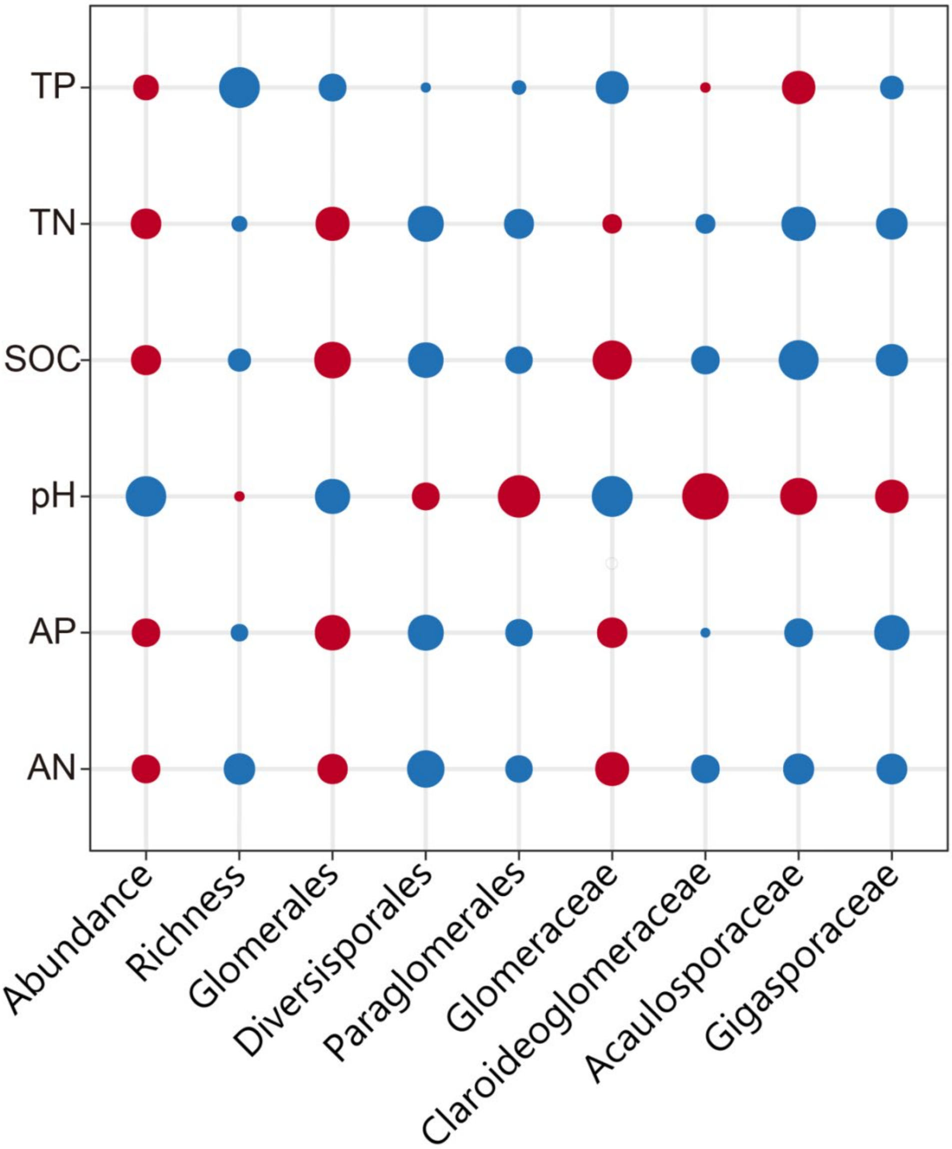

Figure S1: Random Forest heatmap indicates relationships of AMF index and soil properties. The size of circles corresponds to the variable importance; blue and red depict negative and positive Spearman correlations, respectively.

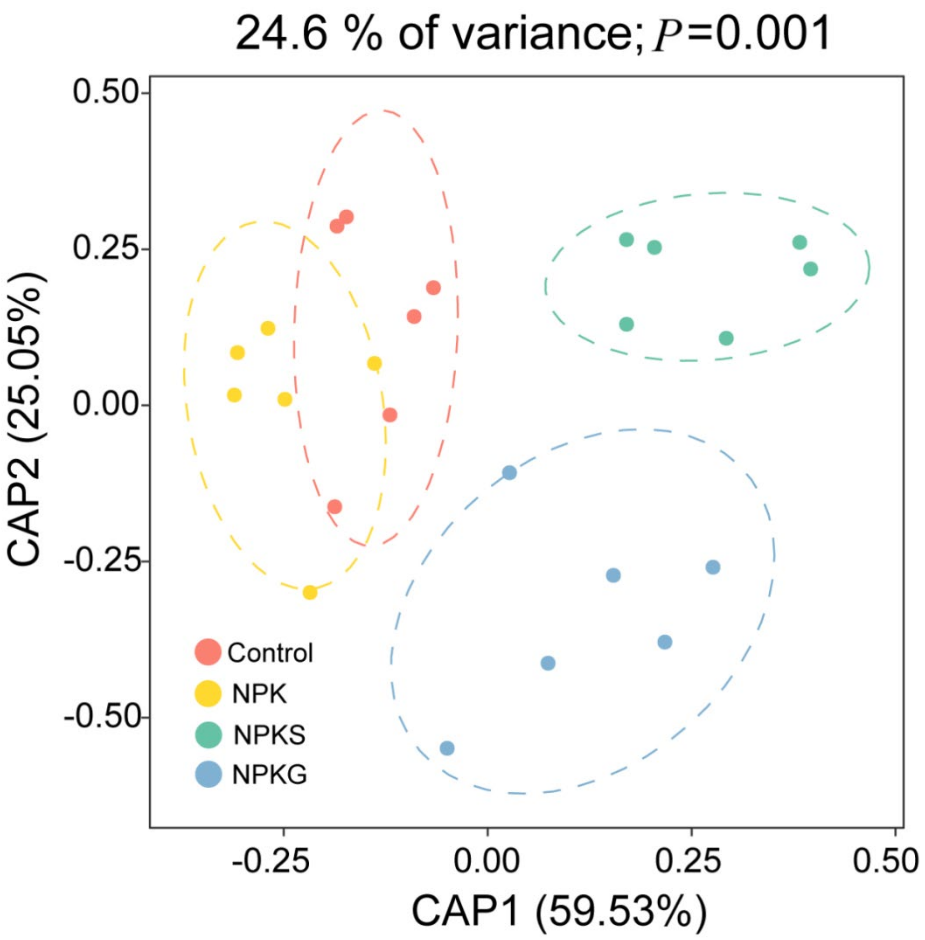

Figure S2: Canonical analysis of principal coordinates (CAP analysis) showing the effects of different practices on soil AMF community composition.

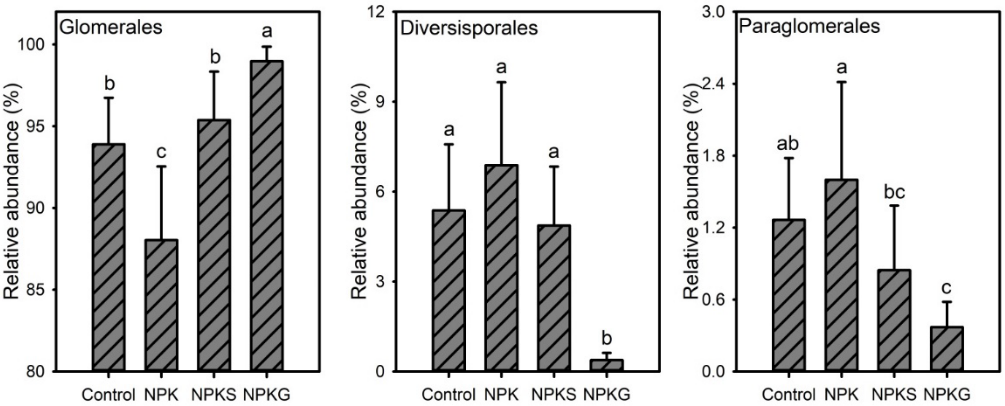

Figure S3: Relative abundance of dominant AMF orders across treatments. Error bars denote standard deviation; different letters represent significant differences from Duncan comparisons (P <0.05).

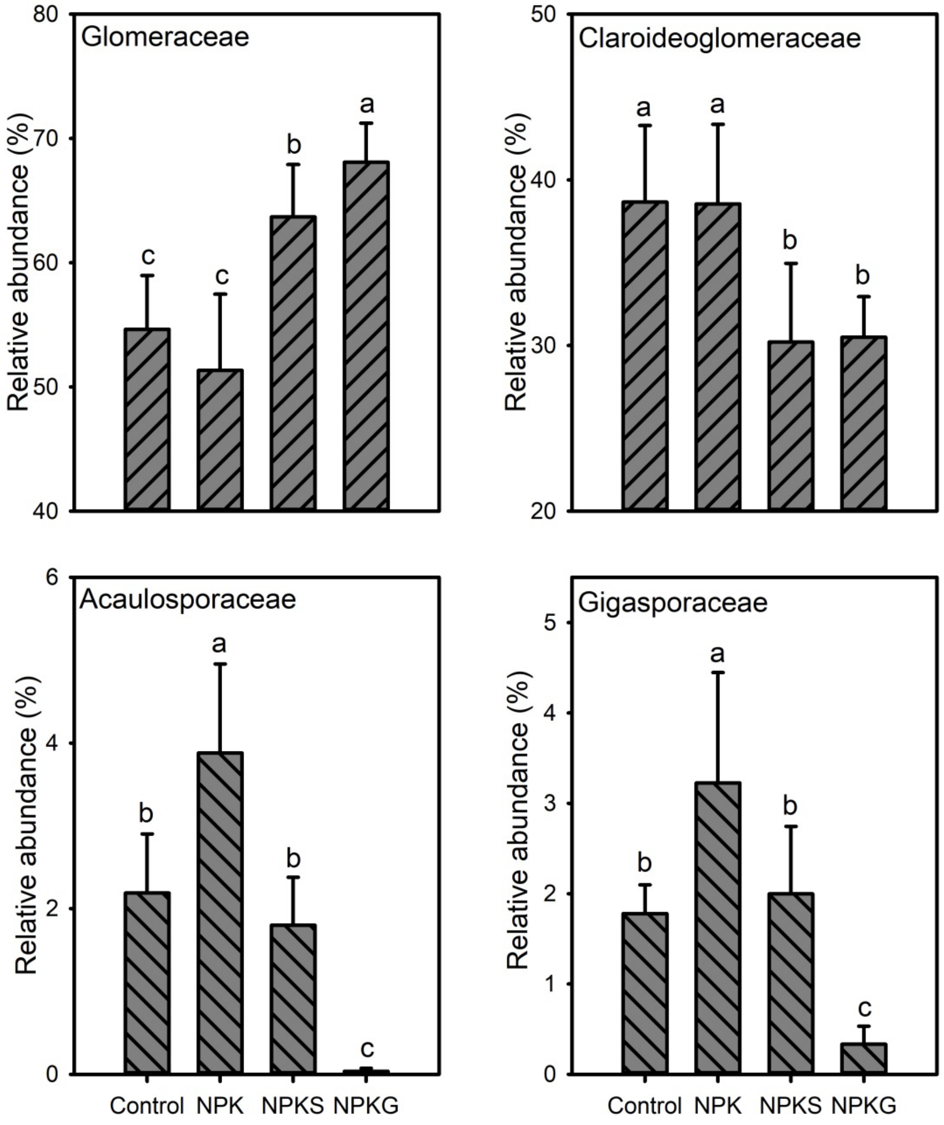

Figure S4: Relative abundance of dominant AMF families across treatments. Error bars denote standard deviation; different letters represent significant differences from Duncan comparisons (*P* <0.05).

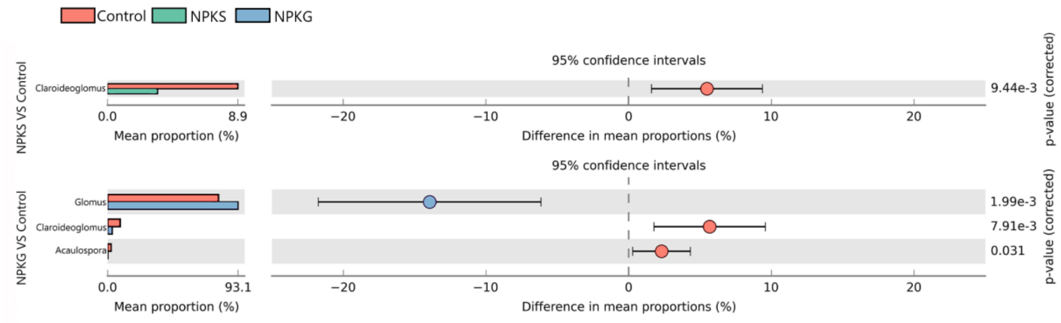

Figure S5. STAMP analysis showing the enriched AMF genera in each treatment.

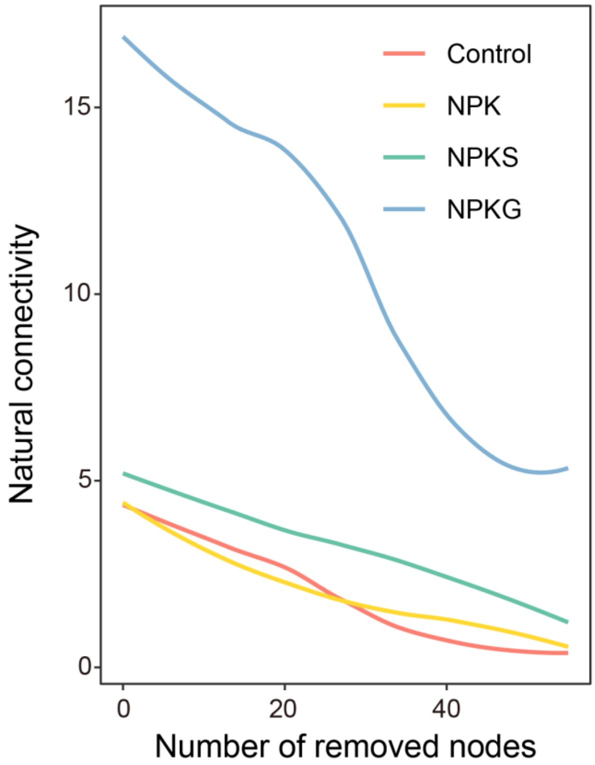

Figure S6: The relationship between the numbers of removed nodes and natural connectivity of AMF co-occurrence network in each treatment.

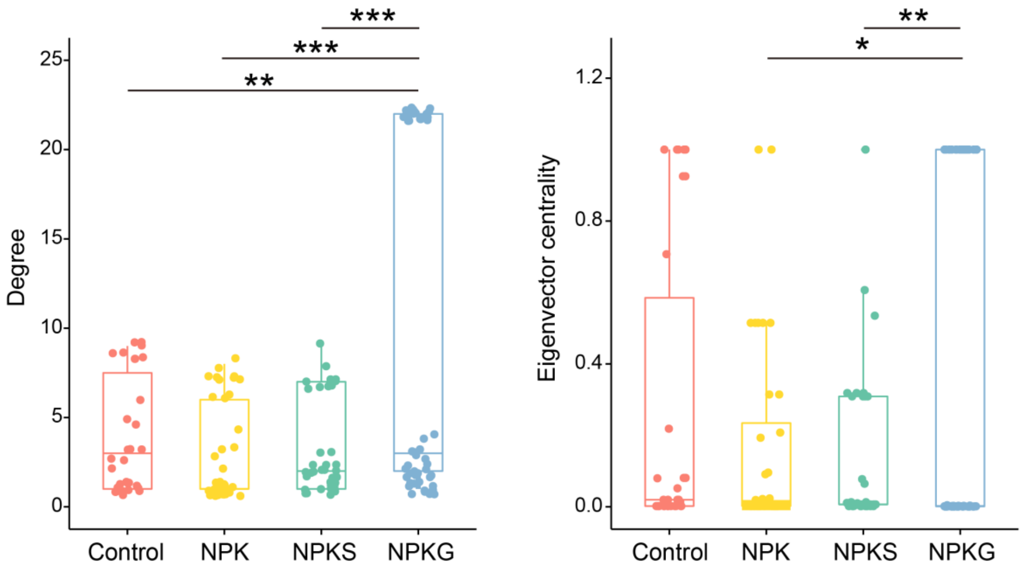

Figure S7: Differences in degree and eigenvector of AMF sub-networks within Glomeraceae under different treatments. *, ** and *** in the figure means significance at the probability of 0.05, 0.01 and 0.001 level, respectively.
